# Computer-Assisted and Data Driven Approaches for Surveillance, Drug Discovery, and Vaccine Design for the Zika Virus

**DOI:** 10.3390/ph12040157

**Published:** 2019-10-16

**Authors:** Subhash C. Basak, Subhabrata Majumdar, Ashesh Nandy, Proyasha Roy, Tathagata Dutta, Marjan Vracko, Apurba K. Bhattacharjee

**Affiliations:** 1 Department of Chemistry and Biochemistry, University of Minnesota, Duluth, MN 55812, USA; 2 University of Florida Informatics Institute, Gainesville, FL 32601, USA; subho@research.att.com; 3 Centre for Interdisciplinary Research and Education, Kolkata 700068, India; anandy43@yahoo.com (A.N.); proyasha.roy@gmail.com (P.R.); tathagata.dk@gmail.com (T.D.); 4 National Institute of Chemistry, Hajdrihova 19, Ljubljana 1000, Slovenia; marjan.vracko@ki.si; 5 Biomedical Graduate Research Organization, Department of Microbiology and Immunology School of Medicine, Georgetown University, Washington, DC 20057, USA; ab3094@georgetown.edu

**Keywords:** viral epidemics, Zika virus (ZIKV), SIR models, 2D graphical method, peptide vaccine design, mahalanobis distance, principal component analysis (PCA), neighborhood matrix, computer-assisted anti-Zika drug discovery

## Abstract

Human life has been at the edge of catastrophe for millennia due diseases which emerge and reemerge at random. The recent outbreak of the Zika virus (ZIKV) is one such menace that shook the global public health community abruptly. Modern technologies, including computational tools as well as experimental approaches, need to be harnessed fast and effectively in a coordinated manner in order to properly address such challenges. In this paper, based on our earlier research, we have proposed a four-pronged approach to tackle the emerging pathogens like ZIKV: (a) Epidemiological modelling of spread mechanisms of ZIKV; (b) assessment of the public health risk of newly emerging strains of the pathogens by comparing them with existing strains/pathogens using fast computational sequence comparison methods; (c) implementation of vaccine design methods in order to produce a set of probable peptide vaccine candidates for quick synthesis/production and testing in the laboratory; and (d) designing of novel therapeutic molecules and their laboratory testing as well as validation of new drugs or repurposing of drugs for use against ZIKV. For each of these stages, we provide an extensive review of the technical challenges and current state-of-the-art. Further, we outline the future areas of research and discuss how they can work together to proactively combat ZIKV or future emerging pathogens.

*“A sickly season,” the merchant said,*

*“The town I left was filled with dead,*

*and everywhere these queer red flies*

*crawled upon the corpses’ eyes,*

*eating them away.”*

*A Medieval Song about the Plague (http://www.historyofpainters.com/plague_art.htm)*

*“How many valiant men, how many fair ladies, breakfast with their kinfolk*

*and the same night supped with their ancestors in the next world!”*

*Giovanni Boccaccio, Of the Black Death*

## 1. Discovery and Brief History

The Zika Virus (ZIKV) was first isolated in 1947 from a rhesus monkey in the Zika forest of Uganda [1]. In 1952, the virus was detected in humans. It spread through Africa and Asia at first. The first major outbreak of ZIKV infection was reported from the Yap Island in the Federated States of Micronesia in 2007. The World Health Organization (WHO) report [1] provides a brief history of the origin and spread of ZIKV. Serosurvey of humans found ZIKV throughout Africa, Asia, and Oceania. In 1954, there was a report of the first three cases of ZIKV infection in humans in Oyo state, Nigeria [2]. In 1979, Fagbami [3] observed that 40% of the Nigerian adults and 25% of the Nigerian children had antibodies of ZIKV.

The first direct evidence of the presence ZIKV in the Asian continent and the first proof of its transmission by an urban vector was found through the isolation of the virus from *A. aegypti* mosquitoes in Malaysia in 1966 [4,5]. After 11 years of this observation, the first human infections in Asia were discovered in central Java of Indonesia in patients having fever, malaise, stomach ache, anorexia, and dizziness [6].

Regarding the spread of ZIKV from Africa to Asia, Liang et al. [7] put forward an interesting idea about this process based on mathematical modeling. These authors carried out quantitative analysis of ZIKV evolution, nucleotide substitution rate, and the time to the most recent common ancestors (tMRCAs). Based on their results these authors concluded that a “global dissemination of ZIKV spread was likely to have originated from Africa, followed by eastward transmission to south-eastern Asia, Oceania, South America, Caribbean, and Central America.”

Concerning this Africa to Asia spread, these authors observed the following: “Relatively few studies on the origin of the South Pacific Rim lineage had been reported in the literature. The tMRCA for South Pacific Rim was estimated to be 1947 (95% HPD interval 35 to 1966) in our study. Coincidentally, during the Second World War in south-eastern Asia, around 100,000 East and West African soldiers were brought into combat in the Burma Campaign from January 1942 to July 1945. Specifically, the British Empire colonial unit 11th (East Africa) Infantry Division comprised troops from East and West African countries such as Kenya, Uganda, Nyasaland, Tanganyika, and Rhodesia (Burma Star Association—The 11th East African Division). During those three years’ conflict, both sides suffered heavy casualties, including at least 20,000 Japanese soldiers who died because of disease in the battle of Imphal. It is also noteworthy that Thai army was also involved in this campaign and that after the Japanese surrendered, troops were continued to be deployed to the then Malaya. The whole campaign could serve as a possible portal of entry for the transmission of ZIKV from Africa to south-eastern Asia during that wartime period.”

The Zika virus attracted a heightened international attention following huge incidence of microcephaly in neo-natal cases in the Latin Americas in 2015 [8]. Historically, the spread of ZIKV happened in two phases [9]: (a) Cases of ZIKV detected during the pre-epidemic period (1947 to 1999), and (b) case history of ZIKV during 2007 to 2017, respectively. On 2 March 2015 Brazil notified WHO of reports of an illness characterized by skin rash in the northeastern states. From February 2015 to 29 April 2015, nearly 7000 cases of illness with skin rash were reported in these states. All cases were mild, with no reported deaths. Zika was not suspected at that stage, and no tests for Zika were carried out. On 1 February 2016, WHO [1] declared that the recent association of Zika infection with clusters of microcephaly and other neurological disorders constituted a Public Health Emergency of International Concern. It should be mentioned that WHO later relaxed this warning subsequently in the November of 2016.

## 2. Virology

### 2.1. Structure

**Figure 1:**
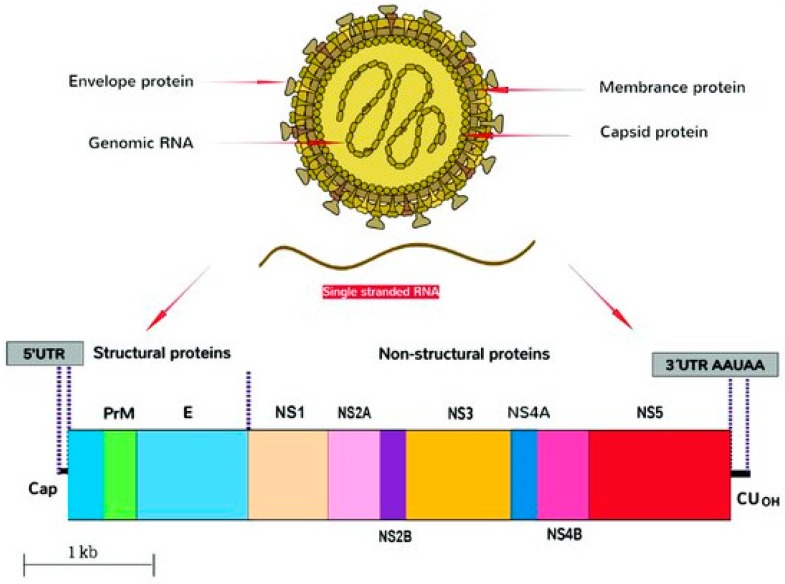
The three structural proteins and seven non-structural (NS) proteins of Flaviviruses. Reproduced under the Creative Commons Attribution License from [12].

ZIKV is a member of the Flavivirus genus that is part of the Flaviviridae family. Some important disease-causing viruses of this genus are Dengue fever virus, Tick-borne encephalitis virus, West Nile virus, Japanese encephalitis virus, and Zika virus [10]. These viruses possess a single stranded positive sense RNA genome containing approximately 11,000 nucleotides [11]. The RNAs of such viruses can be directly translated into a large polyprotein that is subsequently converted by viral and host cell proteases into two sets of proteins: structural proteins and nonstructural proteins. The three structural proteins are: envelope (E), membrane precursor (PrM), and capsid (C) (Figure 1). These are critical for the formation of the capsid and envelop of the mature virus. The seven non-structural (NS) proteins, on the other hand, have important roles in the replication of the virus. The NS group of proteins include: NS1, NS2a, NS2b, NS3, NS4a, NS4b, and NS5 as shown in Figure 1 [9,10,11].

Table 1 summarizes the structure of the Flavivirus genome including the untranslated regions, 5′-UTR and 3′-UTR regions [1,9,10,11].

The 3′ terminal end of ZIKV has a sub-genomic Flavivirus RNA (sfRNA) region that is essential for causing diseases in humans [13,14]. The envelope protein comprises most of the virion surface. It participates in viral-host cell binding and membrane fusion during replication. Replication of ZIKV occurs in the cytoplasm of infected cells [15,16]. After endocytosis mediated by a still unknown cellular receptor, viral fusion occurs with endosomal membranes. This results in the uncoating of the viral particle and release of the viral genome. The genome is subsequently translated into a single polyprotein at the endoplasmic reticulum (ER). The polyprotein is processed by viral NS2B–NS3 protease and other cellular proteases generating mature viral proteins. The positive-sense viral RNA genome is then copied to produce a negative-sense RNA molecule chain, which is used as the template for the synthesis of the full length positive-sense viral genomic RNA. Synthesis of viral genome occurs in association with the ER and is catalyzed by a replication complex consisting of NS5 RNA-dependent RNA polymerase and other NS proteins. The new genomes are packaged by the C protein; they acquire envelopes while budding from the ER. These immature virions thus produced are translocated through the cellular secretory pathways, where E glycosylation and cleavage of prM by host furin protease occurs producing the mature virions that are released by exocytosis.

### 2.2. Evolution and Spread

**Figure 2:**
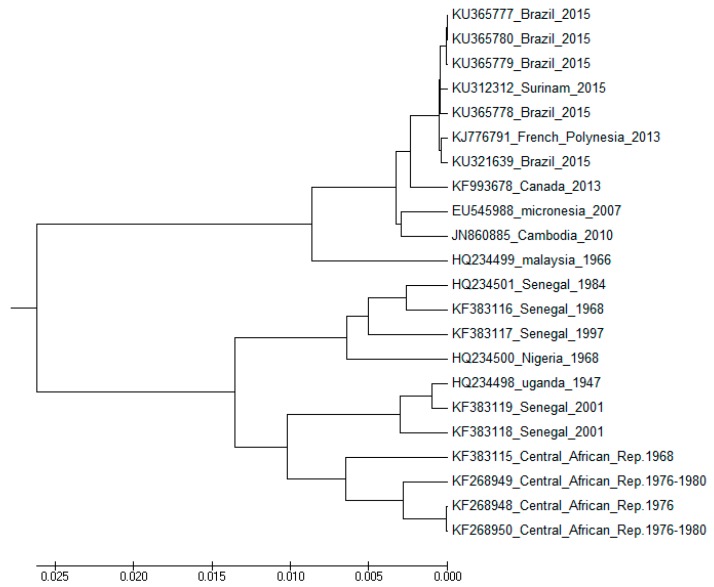
Phylogenetic tree of 22 ZIKV genome sequences [17]. Each sequence is identified with its accession number, country, and year of collection. The horizontal axis shows relative amount of character change between different sequences. Reproduced with permission from Bentham Science Publishers, www.benthamscience.com.

From its initial stage in Uganda, the ZIKV has numerous alterations. Figure 2 gives a phylogenetic tree of 22 ZIKV sequences [17]. There are two major groups: one with older sequences from the African continent and another with more recent Asian, Pacific, and American sequences. The structural differences among these two groups are quantified in detail in [17].

After it was first detected in 1947, the pre-epidemics period of the Zika virus spanned till 1999. Table 2 provides a brief description of this, with year and country-wise details of infection. Following this, a summary of the ZIKV epidemics, from 2007 to the present time, is given in Table 3.

## 3. Infection

### 3.1. Clinical Symptoms and Complications

The Centers for Disease Control and Prevention [20] gives a detailed list of information about the clinical aspects of the Zika virus infection. A majority of people infected with Zika virus do not develop too many overt symptoms. Typical symptoms of Zika virus infection include mild fever, rash, joint/muscle pain, headache, and conjunctivitis. Most people recover from these symptoms in around a week.

ZIKV infection during pregnancy has been linked to several complications, which are collectively referred to as congenital Zika syndrome. These include miscarriage, severe microcephaly, and other fetal brain defects, lasting tissue damage in brain, eye damage [20,21]. Another complication of ZIKV infection is its association with the Guillain–Barré syndrome (GBS), a neurological disorder [22,23].

### 3.2. Modes of Transmission

The current CDC website gives the following information regarding the transmission of the ZIKV transmission [24].

(a) *Through mosquito bites*: ZIKV is transmitted to people primarily through the bite of infected mosquitoes (*A. aegypti* or *A. albopictus*). Mosquitoes become infected when they feed on a person already infected with the virus. Infected mosquitoes can then spread the virus to other people through bites.
(b) *From mother to child*: A pregnant woman can pass ZIKV to her fetus during pregnancy.
(c) *Through sex*: ZIKV can be transmitted through sex from a person who has Zika to his or her partners. The virus can be passed through sex, even if the infected person does not have symptoms at the time. It can be passed from a person with Zika before their symptoms start, while they have symptoms, and after their symptoms end.

This scenario of ZIKV disease transmission is not without precedents in the human history. The first incidences of possibly smallpox in Europe was the Antonine Plague of 165 to 180 AD, also known as the Plague of Galen (from the name of the Greek physician living in the Roman Empire who described it), was an ancient pandemic brought back to the Roman Empire by troops returning from the Near East campaigns [25]. The Plague of Justinian (541–542 AD) afflicted the Eastern Roman (Byzantine) Empire, especially its capital Constantinople, the Sasanian Empire, and port cities around the entire Mediterranean. It probably originated in Ethiopia and came through rodents in ships carrying grain to Rome. Smallpox infections that decimated the native populations of the New World was carried there by the Spanish conquistadores [26]; in turn syphilis was carried eastward from the New World to Europe and thence the whole world. Currently, pandemic viruses gain increasingly rapid circulation through globalization and worldwide fast travels in our inter-connected world.

Human society has been under the constant pressure of epidemics and pandemics from time immemorial. As the 19th century German physician and scientist Rudolf Ludwig Karl Virchow once lamented [27]: Not a single year passes without [which]… we can tell the world: here is a new disease!

## 4. Mathematical/Computational Analysis and Results in ZIKV Virology, Peptide Vaccine Design, and Anti-Zika Drug Design

Currently, there are a broad range of experimental and computational methods available to the scientists and the public health managers worldwide which can be used for the effective management of emerging diseases. In a recent treatise [28], the authors of this review article proposed a “generic approach” of surveillance, mitigation, and vaccine, as well as drug design for emerging pathogens, the four pillars of which may consist of:

(a) Epidemiological approaches for the characterization of reservoirs of next possible emerging pathogens;
(b) Fast computational sequence comparison methods for the characterization of the emerging pathogens to understand how novel or severe they could be;
(c) Once the sequences of the dominant strains have been determined, computer-aided vaccine design (CAVD) methods can be used to produce a set of probable vaccine candidates for quick synthesis/production and testing in the laboratory;
(d) Computer-assisted design of novel therapeutics and testing of new drugs or repurposing already existing FDA-approved drugs.

The remainder of this section discusses and reviews the formulation and use of the above-mentioned multidisciplinary approach for the management of the ZIKV issues.

### 4.1. Quantitative Epidemiological Modelling Strategies to Prevent Zika

The major approaches of quantitative modelling for prevention and mitigation of ZIKV can be broadly categorized into two parts: mathematical models and data-driven statistical/machine learning (ML) modelling techniques. Below we provide outlines of developments in each of these two lines of research.

The well-known susceptible-infectious-recovered, or SIR model forms the foundation of the mathematical models used to predict the spread of Zika. The SIR model is defined by the following differential equations, giving rates of changes at time point *t*, between the three different categories:

(1) $$\frac{dS}{dt}=-\frac{\beta IS}{N},$$

(2) $$\frac{dI}{dt}=\frac{\beta IS}{N}-\gamma I,$$

(3) $$\frac{dR}{dt}=\gamma I.$$

Here β is the disease transmission rate from an infectious person to a susceptible person, and γ is the recovery rate of an infected individual. There has been extensive research on SIR models and their extensions. Its relevance in modelling of several disease spreads, accounting for context-specific priorities like mortality, population growth, vector-borne transmission etc. can be found in [29]. In the case of Zika, a number of works on SIR modelling of the spread of ZIKA are based on estimating its infectious effect. This is obtained through a quantity called basic reproduction number, generally denoted by R_0_. This is defined as the average number of infections caused by an infected person among the population which is susceptible to a disease. In case of ZIKV, the different modes of transmission, viz. vector-borne and sexual, as well as seasonal and geographic variations need to be accounted for while calculating R_0_. To this end, [30] incorporated seasonal patterns in mosquito populations, [31] separately computed R_0_ for different geographical regions, and [32,33,34] extended the basic SIR model to incorporate the sexual transmission as a second mode of infection. A few other approaches can be found in [35] and Table 1 of [36].

One example of Zika-specific extended SIR models is the SEIR model of [30] with separate populations for humans (h) and mosquito vectors (v) with time-varying transition rates. They define a separate exposed (E) category for humans and mosquitos separately, and assume the differential equations for humans and mosquitos:

(4) $$\frac{dS_{h}}{dt}=-\lambda_{h}\left(t\right)S_{h},\frac{dE_{h}}{dt}=-\lambda_{h}\left(t\right)S_{h}-\nu_{h}E_{h},\;\frac{dI_{h}}{dt}=\nu_{h}E_{h}-\gamma_{h}I_{h},\;\frac{dR_{h}}{dt}=\gamma_{h}I_{h},$$

(5) $$\frac{dS_{v}}{dt}=h_{v}N_{v}-\lambda_{v}\left(t\right)S_{v},\frac{dE_{v}}{dt}=\lambda_{v}\left(t\right)S_{v}-\nu_{v}E_{v}-\mu_{v}E_{v},\;\frac{dI_{v}}{dt}=\nu_{v}E_{v}-\mu_{v}I_{v}.$$

Here $\lambda_{h}\left(t\right)$ and $\lambda_{v}\left(t\right)$ are the time-varying rates of mosquito-to-human infection and mosquito birth, respectively, and other parameters are the respective class transition rates. Their numerical results show that there is considerable temporal variation in mosquito incidence and biting rate that the basic SIR model does not capture, and showed through retro-fitting on past data that a seasonal model accounting for variable biting rate can accurately predict future disease incidences.

Data-driven modelling strategies for countering Zika have been explored keeping a diverse types of end goals in mind. In a departure from traditional epidemiological modelling, [37] attempted to predict new primate reservoirs of ZIKV in the wild based on their phenotypical characteristics. This has the potential to help prevent future explosive spreads of the virus through proactive surveillance and prevention of ZIKV spread from the at-risk primate species. A number of studies have explored quantitative structure activity relationship (QSAR) approaches for developing drugs for the treatment of Zika. These include identifying the novel compounds that are inhibitors of different enzymes present in ZIKV [38,39,40], as well as repurposing existing drugs used in treating diseases similar to Zika [41]. A more detailed list of such in-silico methods is available in [42], and are discussed later in this review.

### 4.2. Computer-Assisted Peptide Vaccine Design for Zika Virus

Vaccination is the most effective way to induce an immune response against a pathogen attack. As of now, effective vaccines are available for only a handful of infectious diseases. In this section, we will look at the current methods of vaccine development and particularly, Zika virus vaccines that hopefully might reach the market within the next 2–3 years. Additionally, we will also present a novel technique to design vaccines at the preliminary stages.

Vaccines are developed using either the whole organism, parts of the organism, or modified versions of it. Their injection is able to elicit a weak immune response that is also impactful in creating memory cells that remain in the host bloodstream. In a subsequent naturally acquired infection by the same pathogen, the memory cells will be able to produce a rapid and strong immune response in order to eliminate the foreign agent from the body.

Live attenuated vaccines (LAV) consist of the whole pathogens that are weakened by serially diluting the wildtype pathogen in vitro [43], selectively growing in conditions that reduce the virulence of the pathogens [44] or by introducing single nucleotide mutations. Entire pathogens that have been inactivated, i.e., their proliferative mechanism removed, by means of chemicals like detergents, heat or radiation also serve as vaccine candidates. Surface proteins of the causative agents are also potent in eliciting immune responses [45]. The latest addition to the repertoire of vaccine development are genetically engineered vaccines which include constructs of multiple target and non-target organisms.

In the Flaviviridae family, vaccines are available for the yellow fever virus (YFV) and Japanese encephalitis virus (JEV). There are two types of JEV vaccine – a live attenuated vaccine SA14-14-2 [46] and a genetically engineered vaccine in which two surface proteins are recombined with a weakened YFV strain [47]. None exist for the Zika or Dengue viruses.

An important point to note is that vaccines, especially live attenuated or inactivated, are effective in individuals with fully functioning immune systems and may not work in immunocompromised individuals.

The WHO reports 14 vaccines that are currently under development at Clinical Phase Trial I and 2 vaccines that have moved in to Clinical Phase Trial II [48]. They are either DNA-based vaccines or inactivated whole Zika virus (ZIKV) vaccines. Additionally, there are two recombinant viral vectors, a peptide vaccine based on mosquito salivary proteins and another that utilizes prM-E (pre-membrane-envelope) mRNA transcript of ZIKV. The peptide vaccine is particularly of importance as it aims to provide immunity against a broad-spectrum of mosquito-borne viruses along with ZIKV.

However, one of the challenges faced in administering Zika vaccines is a naturally occurring phenomenon called antibody dependent enhancement or ADE. Reportedly, in cases of a secondary Dengue infection, it has been seen that the disease severity is amplified and a probable cause is an abnormal behavior of the antibodies against Dengue virus—they aid in the virulence of this arbovirus resulting in Dengue Shock Syndrome or Dengue Hemorrhagic Fever [49]. Since Zika is closely related to Dengue, an individual with a Zika vaccination might be severely affected by ADE if she or he is subsequently or naturally infected with the Zika virus.

Despite the advancement of vaccine over the last century, there are still significant logistic hurdles in the developmental process that need to be refined including preserving the longevity of the attenuated live pathogens, sustaining the immunogenicity of the strains that have been rendered inactive, maintaining the integrity of the carrier proteins without interfering in the immunogenic property of the purified polysaccharides, and the possibility of relapse of vector-based vaccines leading to unforeseen genetic response; these are some of the primary challenges that are met in the vaccine production industry. A major setback posed is by the short efficacy of one of the largest productions of antiviral vaccine—the annual flu vaccines. Because of the rapidity and high frequency in the genetic mutation in RNA viruses, flu shots need to be manufactured every year incurring massive production and distribution costs. Hypothetically, a Zika virus would mutate at the rate of 0.12–0.25% [50] every year which may necessitate the annual manufacture of a LAV.

#### Peptide Vaccines

The newest addition to the repertoire of vaccinology are peptide vaccines which encompasses the application of informatics in life sciences like bioinformatics and immunoinformatics in designing individualized vaccines. Essentially, epitopes from conserved regions among proteins with high solvent accessibility are selected for the antiviral vaccine. Such epitopes are then tested for population HLA sensitivity and autoimmune risks. Finally, rank-based epitopes are chosen for further experimental analyses like efficacy, longevity, range, side effects, etc. Based on this workflow, there has been a number of research articles that have predicted a variety of suitable and effective epitopes observed in the capsid, envelope, NS2A, NS3, NS4B, and NS5 proteins of Zika virus [51,52,53,54,55,56].

Addressing the issue of ADE, Dos Santos Franco et al. (2017) [57] were able to specifically elicit the cell-mediated immune response by constructing a NS5-based peptide vaccines that targets both Zika as well as Dengue viruses. Furthermore, Richner et al. (2017) [58] assembled modified prME mRNA of Zika virus in lipid nanoparticles. The genetic modification allowed for the inactivation of a cross-reactive envelope protein epitope that led to decreased development of cross-reactive antibodies.

In order to detect the effective epitopes for peptide vaccine design, the first step is to scan the target protein sequence for segments that are of acceptable length and effective in provoking an immune response. Nandy et al. (2009) [59] proposed a reliable graphical method to glean such sequence information. Their approach includes assigning each of the 20 amino acids to an axis on a 20-dimensional grid. For a sequence, starting with the first amino acid, a point is plotted in the corresponding direction. For the subsequent amino acids, a step is taken toward the corresponding axis, thereby, tracing a graph in its wake that represents the sequence composition and distribution. For each graph, a descriptor or a graph radius is defined as *pR*, which is the distance measured between the origin and the weighted center of mass (c.m.). The weighted c.m. is the weighted average of the coordinate values in each direction and is expressed in Equation (3).

(6) $$\mu_{1}=\frac{\sum_{i}x_{1i}}{N},{\;\mu }_{2}=\frac{\sum_{i}x_{2i}}{N},\;\ldots ,{\;\mu }_{20}=\frac{\sum_{i}x_{20,i}}{N}$$

(7) $$p_{R}=\sqrt{\mu_{1}^{2}+\mu_{2}^{2}+\ldots +\mu_{20}^{2}}$$

where $x_{ji}$ is the coordinate of the *j*-th amino acid, *j* = 1, 2, …, 20, *i* = 1, 2, 3, …, *N*, *N* being the length of the protein sequence and *pR* the graph radius from the origin to the center of mass. The value of *pR* is immediately seen when comparing two or more sequences of any length. It is a reliable sequence characteristic with identical sequences having the same *pR* values. Setting a scanning length of 10–14 amino acids, the *pR* is determined for each segment of the sequence by moving the scanning window at each amino acid position. Thus, for a given protein sequence, and based on the moving window length, multiple *pR* values will be obtained. When searching through a more exhaustive list of sequences, the moving window will check for the average *pR* for segments of 10–14 amino acid in length. A profile of *pR* values will be obtained for each position in the protein sequence, indicating the degree of variability in a region. The higher the conservation, lower the amino acid variability. It must be noted that the *pR* cannot distinguish between synonymous mutations. Therefore, the degree of conservation is in terms of identical amino acids occurring in a particular position. The simplicity of this method allowed the authors to predict well conserved peptide regions for over 500 H5N1 neuraminidase proteins and in excess of 400 H1N1 neuraminidase proteins [60]. Furthermore, they were also successful in predicting epitopes among 438 rotavirus VP7 surface glycoprotein and for more than 200 sequences of HPV L1 capsid protein [61].

Subsequently, the conserved peptide regions are superimposed on an average solvent accessibility profile of the same protein. Conservation at the highest levels of solvent accessibility are ideal candidates to move onto the next step of the rational design of peptide vaccines. Based on the myriad bioinformatics tools and servers at our disposal, and based on the HLA population of the target vaccination community, the selected peptides are determined for strong immunogenic response. Next, a rudimentary BLAST search for sequence similarity will eliminate any peptide candidate likely to cause an autoimmune response. With this exercise, the conserved peptides with high solvent accessibility and no autoimmune threats are to be tested for in vitro and in vivo analyses. Dey et al. (2017) [62] were able to identify and characterize four Zika virus epitopes in the envelope protein for an effective vaccine design.

Despite the varied advantages offered by peptide vaccines, the requirements for adjuvants and carrier proteins, and the low intensity of immune response among many others [63] have curbed the advancement of peptide vaccines reaching human clinical trials. On the other hand, in a 2006 study by [64], it was observed that mice which had been previously exposed to a virus, died during the second exposure to peptide vaccines derived from the original virus because of over-stimulation of antibodies and excessive cytotoxicity levels by T-cells. Thus, an optimum dosage is critical for peptide vaccines. Some of this slack is being taken up by an exciting new approach to vaccines—in silico antibody design by reconstructing antibody structures through parts of other antibody structures available in the databases which augments the antibody effectiveness manifold. This is discussed in a later section (Section 4.4.2) of this review.

New developments in bioinformatics, information technology, and immunoinformatics are poised to take the science of vaccine design to new heights. The best responses to vaccines will be from those designed specifically for an individual at a specific time period of his/her life—clearly an impossible task at the current level of technology. However, there are possibilities of using artificial intelligence (AI) to scan all the genes taking part in the human immune response, especially the adaptive receptors on human B and T immune cells, over millions of individuals to model the human immune response and thereby discover and engineer precision vaccine design [65]. Use of AI will signal a shift from hypothesis-driven science to discovery-driven science. IBM Watson is reported to be already more efficient in detecting cancer symptoms than humans; precision vaccine design by AI may not be too far a goal [66].

The Zika virus pandemic has eased considerably after the sudden eruption in South America in 2015–16 except for sporadic infections around the globe such as in Ahmedabad, India in 2017 [67]. However, this quiescence should not be taken as a sign of the end of the epidemic since dormant viruses have been known to resurge at opportune moments. The experience of space travel has shown that many dormant viruses reactivate during the space travel and can lead to severe physiological disorders such as compound organ infections and organ failures, believed to arise from the severe stresses of space travel [68]. At a more down-to-earth level, there can be extreme stresses experienced by the people especially in poor, underdeveloped, and developing countries, which may lead to a resurgence of viruses that are relatively dormant at the present time. Surveillance and monitoring of the viruses in the wild as well as continuing research in development of drugs and vaccines therefore should be conducted at as much an urgent level as possible under the circumstances.

### 4.3. Use of Sequence (Structure)-Property Similarity Principle and Alignment Free Sequence Descriptors in the Characterization of ZIKV Sequences

*“All cases are unique and very similar to others.”*

*T. S. Eliot, The Cocktail Party*

As indicated in Section 1 above [1,9,17,27], Zika virus is an evolving pathogen undergoing numerous changes during its transmission from its site of origin (Uganda) to the countries of Africa, Asia, and the Americas; according to our sequence descriptor index, explained later in this section, an estimate of the overall change in genome sequences would be about 11.63% from Africa to Asia, and another 4.55% change from Asia to the Americas. When a viral pathogen suddenly emerges, it is important that we compare its sequence with already known strains of the same organism and try to assess its degree of novelty and probable pathogenicity/pandemicity as early as possible. The alignment-based methods like BLAST [69] are popular for the comparison of sequences. In the comparison of chemical structures, there are alignment-based methods like comparative molecular field analysis or CoMFA [70,71] and alignment-free methods based on holistic molecular descriptors [72,73,74] which quantify various aspects of the entire molecular structure. Some studies on the comparison of CoMFA and numerical mathematical descriptors for chemicals show that such methods give comparable results [72,73,74]. In a similar fashion, the field of bioinformatics also witnessed the development of alignment-free sequence descriptors [75,76]. Such alignment-free numerical descriptors and factors derived from them may be used as fast tools for sequence comparison for emerging pathogens and gain new insights.

For the comparison of sequences of ZIKV we have used two classes of descriptors based on geometrical and matrix method approaches. In the Nandy et al. [17,75,76] approach, sequences are represented by graphs, G = (V, R), where the bases of DNA/RNA represent the set of vertices V and the inter-base bonds represent the edge set R. On a 2D rectangular grid system one may start plotting a sequence from the origin going one step in the negative x-direction for an adenine, one step in the positive y-direction for a cytosine, one step in the positive x-direction for a guanine, and one step in the negative y-direction for a thymine (uracil). Doing this successively for each base in the sequence in order plots and gives an overview out a graph of the sequence on the 2D grid that displays the base distribution in the sequence.

**Figure 3:**
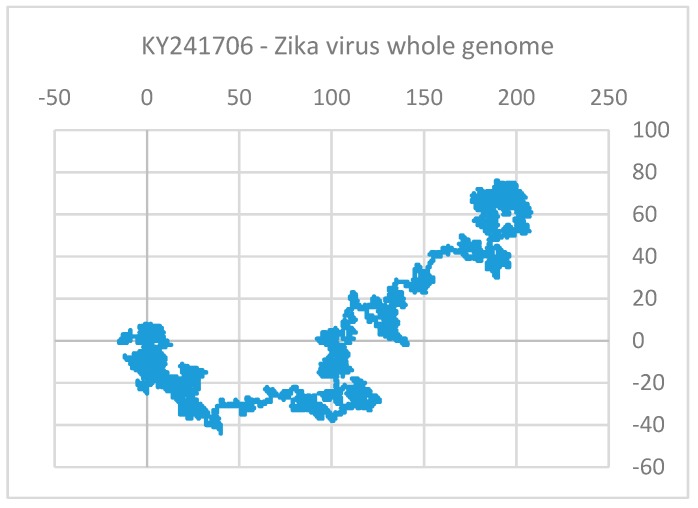
The 2D graphical representation of the Zika virus whole genome (GenBank Locus ID KY241706), methodology as per [75]. The axes are assigned as follows: A (adenine) to the negative *x*-axis, c (cytosine) to positive *y*-axis, g (guanine) to positive *x*-axis and t (thymine) to negative *y*-axis.

Thus, a graphical representation of the Zika virus whole genome sequence would look like the one shown in Figure 3. The figure gives an overview of the base distributions along the whole Zika virus genome. It shows that while the structural genes at the beginning of the sequence have a good homogeneous mixture of the four bases, the non-structural genes that make up the bulk of the genome have a GC-rich texture; it is an overall information we get of a sequence through the 2D graphical representation method. Assigning x and y co-ordinates to each vertex in the 2D graph, one can define the distance, *gR*, from the origin to the center of mass of the plot [77]. The *gR* turns out to be a very sensitive measure of the base distribution in the sequence and therefore is taken as an index, a descriptor of the sequence. Table 4 shows the *gR* values of the Zika virus genomic sequences in Africa, Asia, and South America from selected hosts, all of same length (10272 nt) for easy comparison; the change in *gR* values as we move from Africa to Asia to the Americas indicates (in %) an overall estimate of how much changes in absolute terms have taken place in base distribution and composition of the average genome sequence collected from these continents. It can be seen that the sequence base distribution is becoming more set and compact with time. A phylogenetic tree of Zika virus sequences (Figure 2) also shows clear separation between the three groups, less so between Asia and America than between Africa and Asia as also seen quantitatively through the *gR* values.

In the Vracko and Randic approach [78], DNA/RNA sequences were represented by four neighborhood matrices. The first matrix counts the four bases (A, T, C, G). The next matrix counts the neighboring pairs, AA, AT, AC, AG, TA, TT, TC, TG, CA, CT, CC, CG, GA, GT, GC, and GG while the next two matrices consider the second and third neighbors. Further details of this representation and the application are given in [78].

#### Clustering and Analysis of ZIKV Sequences

Clustering (grouping) and classification (categorization) of objects are two fundamental principles often applied in various fields of research. In such approaches, one collects the objects, which are in “some manner similar” and orders them into categories expecting that the objects in the same category possess in “some manner similar” properties. In the case of ZIKV, the objects are RNA sequences, which encode the structure (sequence) of the virus, identified from different areas of the globe (Africa, Asia, South America). Comparison of ZIKV sequesters using calculated sequence descriptors follows the sequence (structure)-property similarity principle [79].

Sequence descriptors can be used in conjunction with established methods such as principal component analysis (PCA) and self-organizing map (SOM) for sequence comparison [80]. The basic idea of SOM or Kohonen neural network is the projection from multi-dimensional object space onto two-dimensional grid (map) of neurons. Mathematically, the neurons are multi-dimensional vectors. The projection or learning of network is an iterative non-linear algorithm that arranges samples in two-dimensional map with respect to the similarity among them [80]. Because of the basic property of the mapping to locate similar objects, depending on how they are represented using sequence descriptors, close to each other the Kohonen and CP-ANN are inherently useful tools for clustering. The analysis of the SOM map enables us to recognize the clusters and thus the similarity relationships within the data.

**Figure 4:**
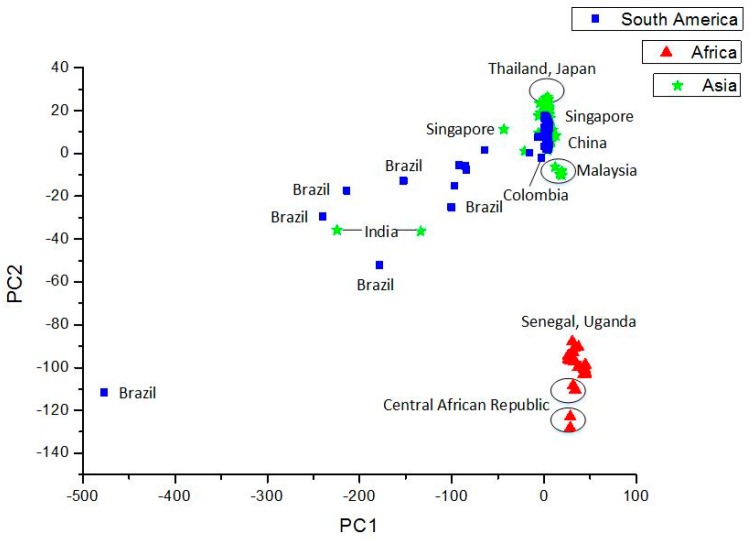
Principal component analysis (PCA) score plots of ZIKV strains from different continents.

**Figure 5:**
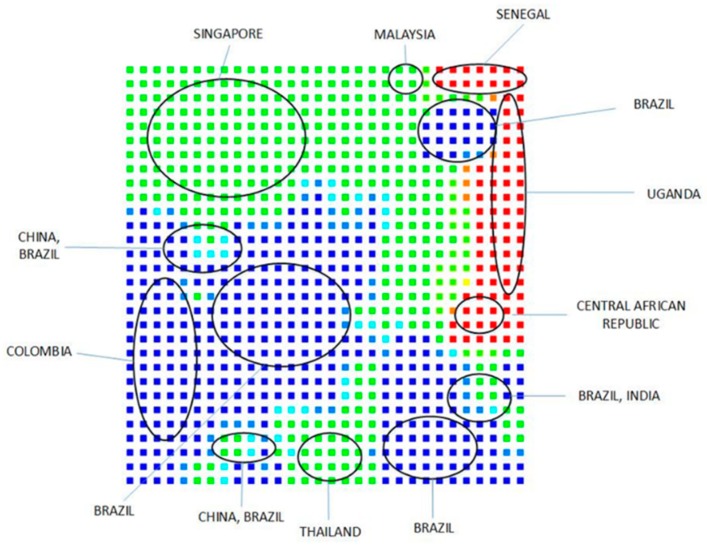
Distribution of objects in self-organizing map (SOM). Red, green, and blue areas represent Africa, Asia, and South America, respectively. Magenta represents overlap between Asia and South America and orange between Africa and Asia.

Following the standard methodology used in previous studies [80], Figure 4 and Figure 5 show the results of PCA- and SOM-based clustering analysis using the orthogonal descriptors for ZIKV sequences [81]. For PCA and SOM calculations, the standard MATLAB program package and in-house developed software were applied, respectively [82]. The data set consists of 310 genome sequences of ZIKV hosted in different species (83% in Homo sapiens, 17% in Simiiformes, Aedes, Macaca or tissue culture cells), and 53 sequence neighborhood descriptors. Most of the genomes with less bases stem from Africa, perhaps because of the fact that the Africa samples are older than Asia and South America ones. Further details are available in [81].

An alternative method for clustering Mahalanobis distances to the center of mass can be calculated. The Mahalanobis distance *d* of an object *a* to the center *b* is defined as [83]:

(8) $$d\left(a,b\right)=\sqrt{{\left(a-b\right)}^{T}V^{-1}(a-b}),$$

*V*^−1^ being the inverse of covariance matrix. The metric of the space is no more Euclidian but is defined by the distribution of objects in the representation space. In other words, the information about the data set is compressed into the space metric. In statistics, the Mahalanobis distance represents an important tool to show how good an individual object fits into the existing data set, or data domain. In our analysis with 310 samples, the 18 outliers, i.e., the objects with larger distance than average + 2*standard deviation are shown in Table 5.

### 4.4. Discovery of Potential Anti-ZIKV Drugs from Literature including Computer-Assisted Approaches

*“Computers are incredibly fast, accurate, and stupid.*

*Human beings are incredibly slow, inaccurate, and brilliant.*

*Together they are powerful beyond imagination.”*

*Albert Einstein*

ZIKV is a flavivirus family of infection transmitted by mosquitos. Because of a series of recent outbreaks in South America, particularly in Brazil before the 2016 Olympic games, it became a serious pandemic situation. The concern for the infection heightened because of babies born from ZIKV infected mothers showed microcephaly and neurological complications [84]. Since neither any drug nor any vaccine is currently available for the treatment and prevention of this infection, potential counter measures are extremely necessary. In addition to a recent review contribution in the form a book chapter on anti-ZIKV drug discovery [85], this section presents several other potential anti-ZIKV compounds from literature including author’s own computer-assisted efforts toward the pursuit.

With ever changing technologies, drug discovery has become a complex process. It requires ten to fifteen years for taking a new molecule from the bench of discovery to the shelf of a pharmacy for human use. The procedure has to undergo several stringent rounds of animal testing, toxicity evaluations, and human clinical trials before approval by the FDA. Current estimated cost of the process is approximately two billion US dollars. Thus, newer technologies are highly valuable for the pharmaceutical industry to improve the efficiency of the process [86]. Emergence of in-silico technologies has proven remarkable success in many frontiers of this procedure over the past few decades including pharmacophore modeling and virtual screening of compound databases to identify potential new drugs [87].

However, it is fundamentally important to understand how a molecule becomes a drug molecule. Idea of lock and its key is the mechanism behind understanding the concept. When a molecule can optimally bind to the active site of a diseased receptor protein or enzyme to trigger or inhibit its biological response, it has the potential to become a drug molecule for that disease. This concept is also the basis of the idea of pharmacophore in drug discovery. By definition a pharmacophore is “an ensemble of steric and electronic features that are necessary for optimal interaction with a specific receptor target structure (a protein or an enzyme) to trigger or inhibit its biological response” [88]. Therefore, stereoelectronic properties of a potential drug molecule must optimally interact with the corresponding properties at the active site of the diseased receptor molecule (protein or enzyme) to trigger or inhibit the specific biological response. Study of stereoelectronic properties of bioactive molecules should therefor provide valuable information in understanding this intrinsic “interaction pharmacophore” necessary in order to discover a drug molecule for a disease [89]. For accurate estimate of stereoelectronic properties, quantum chemical (QM) methods are the best possible choices despite being more time consuming. The stereoelectronic profiles generated from the property calculations of molecules are known as the “interaction pharmacophores”. Although no anti-Zika drug is currently available, several promising ZIKV drug targets have been reported in recent years [90]. These targets with compounds have shown potent activity against ZIKV and its proteins. Non-structural (NS) flavivirus proteins are believed to play important roles in both replication and virion maturation [91]. Crystallographic structures of several ZIKV related NS proteins are now available from protein structural data banks [92]. The NS proteins from the data bank are primarily useful for performing computational studies and are also important for experimental studies. These proteins include NS1, NS2B–NS3 protease, NS3 helicase, NS5 methyltransferase, NS5 polymerase, NS5 full-protein and envelope glycoprotein. Importantly, three of these NS proteins, NS2B–NS3 protease, NS3 helicase, and NS5 methyltransferase are available with ligands, ATP and RNA in the data bank. Thus, both identification of ligand-binding-site for virtual screening and pharmacophore modeling for discovery of potential anti-Zika drugs have been highly facilitated. Pharmacophore models significantly contributed to the discovery of drugs in the past years for retrieving inhibitors against various drug targets [85].

Various approaches have been pursued to identify compounds against ZIKV, targeting host proteins including repurposing of FDA approved drugs [93], high throughput screening [94], phenotypic screening [95], and computational methods [42,96]. As a result, several potential small molecules, vaccines, and therapeutic antibodies have been identified and developed having anti- ZIKV activity. However, most of these studies were in vitro analysis and only a few selected 20 compounds were actually evaluated in vivo. Promisingly, three compounds of these compounds could reach phase I clinical trials [90]. Subsequently, many promising leads including nine vaccines reached phase I or II clinical trials against ZIKV [97]. However, none of these compounds has yet been approved by the FDA profiles [89].

#### 4.4.1. Potential Anti-Zika Targets

A list of anti-ZIKV compounds discovered in recent years with the potential to be drugs for ZIKV is presented in Table 6.

In addition, the following important targets that have been frequently implicated for potential discovery of ZIKV drugs and are thus further discussed below.

*Viral proteins as ZIKV drug targets*: Viral glycoproteins play important roles for virus infection and replication processes as it is involved with virus adsorption, internalization, and fusion with the host cell, as well as with the development of neutralizing immunity. In general, small molecules interfering with the function of any ZIKV viral proteins should have the potential to restrict virus replication and prevent from progress of ZIKV related pathogenesis and diseases. Envelope glycoprotein inhibitors too are well documented for anti-ZIKV activity [114]. Several inhibitors targeting viral proteins that have been identified from high-throughput cell-based screening, in-silico docking, and compound library screening. Some of them have even undergone in vivo testing, for example, EGCG (epigallocatechingallate), a polyphenol present in green tea has exhibited inhibition to the entry of ZIKV into the host cell [114].

*Protease inhibitors*: ZIKV being a flavivirus also possesses the NS2B-NS3 protease. Many natural products have been tested against NS2B–NS3 protease and surprisingly, some of them were found to inhibit ZIKV protease activity [108,115].

*NS3 helicase inhibitors*: These inhibitors bind to the RNA and ATP binding sites [109]. Although a few NS3h inhibitors are reported in the literature, they are mostly identified using computational methods, mainly through virtual screening of compound databases, but none are experimentally validated [116].

*NS4B inhibitors*: Very few studies on ZIKV NS4B protein inhibition are performed so far, mainly because of poor ADME properties of the identified inhibitors. Search for NS4B inhibitors faces many challenges, such as poor drug-like properties of the inhibitors and difficulties in finding pan-flaviviral inhibitors avoiding risk of developing resistant viruses [117,118,119].

*NS5 methyltransferase inhibitors*: The structure of ZIKV MTase with the S-adenosyl-L-methionine (SAM) analog, sinefungin was recently elucidated. In the structural elucidation, Hercik et al. proposed that designing of an inhibitor connecting sinefungin with a Cap analog through a linker could provide stronger affinity to the protein and can make it a more potent compound [111]. Based on this hypothesis, Stephen et al. performed virtual screening of a database comprising about 20,000 compounds and shortlisted ten compounds for experimental evaluation. Four out of these ten compounds exhibited experimental viral growth inhibition below 20 mM concentration with one inhibitor showing IC50 value of 4.8 mM [120]. However, designing ZIKV-specific MTase inhibitors remains a challenge, mainly because of the selectivity of inhibitors to the SAM-binding site, presence of SAM-utilizing proteins in the host cell, and similarity between the human RNA and DNA MTases [121]. In addition, competitive MTase inhibitors in high cellular SAM concentration may cause difficulty in the design of such inhibitor [122].

*NS5 polymerase inhibitors*: A few nucleoside inhibitors, such as 2′-C- and 2′-O-methyl-substituted nucleosides, 2′-C-fluoro-2′-C-methyl-substituted nucleosides, 3′-O-methyl-substituted nucleosides, 3′-deoxynucleosides, derivatives with a 4′-C-azido substitution, heterobase-modified nucleosides, and neplanocins were found to be potent when tested against ZIKV. The most promising inhibitors were the 2-C-methylated nucleosides with IC50 values <10 mM [110].

*Host proteins as drug targets*: Since ZIKV like other flaviviruses contains a small genome, the host cell machinery will have to carry out the core functions that are essential to viral replication. Therefore, inhibition of the viral protein functions could be an attractive target for ZIKV drug discovery. Moreover, broad-spectrum strategy to target host cell processes could also be advantageous as they are often employed by multiple viruses and are less prone to development of drug resistance [123].

*Host cell nucleoside biosynthesis inhibitors*: Nucleoside analogs have been commonly used as antiviral agents for human viral infections for many years [124]. Nucleosides from the host cell are essential for maintaining adequate RNA replications in viruses. Inhibition of nucleoside biosynthesis is believed to trigger the activation of antiviral interferon-stimulated genes in human cells [125]. Ribavirin, one of the first clinically used broad-spectrum antivirals and a few other known broad-spectrum antivirals were found to inhibit the virus-induced cytopathic effects (CPE) of several flaviviruses, including ZIKV (EC50 = 142.9 mg/mL) [126]. However, poor inhibition of ribavirin as shown in the above virus-induced CPE study indicates that it is not a suitable candidate as a ZIKV drug [111]. Other tested nucleoside biosynthesis inhibitors showed better results, such as MPA (EC50 = 0.11 mM), brequinar (EC50 = 0.08 mM), and 6-azauridine (EC50 = 0.98 mM) [127]. However, a recent study indicates several new promising lead candidates for further development particularly as antivirals against ZIKV [127]. BCX4430 an adenosine nucleoside analog has shown EC50 values in the low μg/mL range (3.8–18.2 μg/mL) in vitro, with favorable selective index values and found to be a selective inhibitor of viral RNA-dependent RNA polymerase (RdRp). The compound has also shown in vivo efficacy in AG129 mouse model of ZIKV. Treating the animals with BCX4430 (300 mg/kg/d) protected seven of eight mice from mortality induced by a Malaysian strain (P 6–740) of ZIKV [128]. This protective efficacy of BCX4430 encourages its further development on the basis of safety and better efficacy evaluations. Another RdRp inhibitor, NITD008, an adenosine nucleoside analog, has also exhibited antiviral activity against ZIKV in vitro with EC50 at the sub-micro molar range (0.28–0.95 μM). Although when NITD008 was treated with 50 mg/kg in the A129 mouse model, it protected 50% of the infected mice from death without symptoms of neurological disorder [129], the compound was discarded to other undesirable toxic effects in clinical testing [99].

*Non-nucleoside RNA polymerase inhibitors*: By in silico screening of a compound library of 100,000 small molecules based on Zika RNA-dependent RNA polymerase (RdRp) structure, ten lead compounds were reported in a recent study [130]. Through in vitro tests in Vero cell assays, one of these compounds, 3-chloro-*N*-[(amino)carbonothioyl]-1-benzothiophene-2-carbox-amide (TPB), a non-nucleoside, was found to inhibit ZIKV replication with an EC50 in submicromolar concentration (0.94 μM) [130]. In silico molecular docking evaluations suggest that the compound binds to the catalytic site of the ZIKV RdRp, acting as an allosteric agent to block the viral RNA synthesis. Furthermore, in vivo studies (25 mg/kg) in animals (immunocompetent mice) infected with ZIKV and treating with the compound, TPB showed a reduction of 40-fold plasma viral load compared to the untreated controls. In addition, PK analysis of the compound in the same animal in post-injection period was also found to retain it in the mouse plasma at 150–100 ng/mL level for 10–12 h. Thus, TPB appears to be a promising compound against ZIKV infection [130].

Another non-nucleoside inhibitor, emetine was reported to potently inhibit ZIKV NS5 RNA polymerase activity by binding to its active site. In vitro activity was reported to be in the range of EC50 values of 0.009–0.053 μM. This compound too was reported to be promising in in vivo studies and significantly reduced the levels of both NS1 protein and ZIKV RNA in serum and liver, respectively [131].

*Host cell lipid biosynthesis inhibitors*: Viral infections including dengue and ZIKV are known to be associated with alterations in the lipid homeostasis [132] and disruptions in the membrane structure of infected cells [133]. Cholesterol is found to modulate the host response of several flaviviruses. Although the exact mechanism of this modulation process is yet unclear, inhibition of the biosynthesis pathway of cholesterol could be an attractive therapeutic approach for ZIKV drug discovery.

As a part of cell-based screening of 725 FDA-approved drugs, Lovastatin, an HMG-CoA reductase inhibitor was tested against ZIKV and found to be potent through a dose–response assay (EC50 = 20.7 _ 8.6 mM) [96]. Another related drug, Mevastatin also showed anti-ZIKV activity at concentrations of 1–5 mM, but found not to be dose-responsive above 5 mM [112]. Since Mevastatin is known to induce apoptosis [134], perhaps it showed lack of antiviral activity at higher concentrations.

*Host kinase inhibitors*: Host cell kinases have been documented to play an important in the replication of several RNA virus families [135]. Using a two-step approach of drug repurposing screen by measuring the caspase-3 activity and cell viability with primary hits, Xu et al. were able to discover PHA-690509, a cyclin- dependent kinase inhibitor (CDKi), that inhibited ZIKV infection with an EC50 value of 1.72 mM [94]. Additional 27 CDK inhibitors were also tested by the group to identify nine that could inhibit ZIKV replication. Among these inhibitors, seliciclib (a purine analog, also known as roscovitine) and RGB-286147 were found to inhibit ZIKV infection at sub micro molar concentrations [95]. From these results, the authors remarked that one or more host CDKs might be important for ZIKV replication process as flaviviruses are not known to encode any CDK [91]. Therefore, study on known CDK inhibitors could be useful for discovery of anti-ZIKV therapeutics. It may be worthwhile to mention that an earlier reported malarial CDK (pfmrk) pharmacophore model [136] could be useful for virtual screening from a database of antimalarial CDK (pfmrk) inhibitors to identify potential anti–ZIKV compounds. The CDK pharmacophore model on malaria allowed identification of several new antimalarial CDK inhibitors [136]. Thus, search for additional CDK inhibitors using pharmacophore modeling could be attempted for discovery of potential anti-ZIKV drugs.

Another recent reported study suggested that Axl receptor (a tyrosine kinase, originated from a Greek word anexelekto, meaning uncontrolled) to be a target for discovery of compounds against ZIKV infection [137]. The inhibitors for the receptor seem to function by inhibiting Axl receptor dimerization considered crucial for virus entry. Sarukhanyan et al. experimentally validated the hypothesis by demonstrating anti-ZIKV activities of compounds such as warfarin and a few similar structural analogues identified using in silico methods [106]. Although Axl is thought to mediate viral attachment to the host cells, the absolute mechanism of function is not fully understood [138].

*Protein metabolism disruptors*: Viral replication depends upon the protein metabolism or ER functions, both of which are intimately associated with the intracellular membrane where the viral polyprotein is expressed and processed [139]. However, these processes produce considerable stress on this organelle [139]. So far only one known disruptor, celgosvir (6-O-buta-noyl castanospermine) was tested against ZIKV that showed poor activity in a CPE- based assay in Vero 76 cells infected with either MR766 or PRVABC59 strains (EC50 > 50 mM in both cases) [112].

*Viral entry blockers*: Viral entry blockers, such as saliphenylhalamide (SaliPhe), act by the inhibition of the vacuolar ATPase can also function as anti-ZIKV drug [140]. It was tested in Vero 76 cells, yielding an EC50 value of 0.62 mM for MR766 strain and 0.49 mM for the PRVABC59 strain. Another compound, Niclosamide blocks the acidification of endosomes but probably with a different mechanism that is not yet fully understood [141]. It was identified through screening against ZIKV in SNB-19 cells showing an EC50 value of 0.37 mM based on the measurement of intracellular viral RNA [95]. Incidentally, niclosamide is an FDA-approved drug, mainly used in the treatment of intestinal helminthiasis and also in pregnancy. However, the drug has not undergone important controlled studies in pregnant women. Moreover, the drug failed in the risk assessment study of the fetus in animal reproduction.

#### 4.4.2. Other Approaches

Although no drug has yet been approved by FDA for the treatment of ZIKV infection, numerous small molecules have been identified that have shown anti-ZIKV activity [99,112,142]. However, most of these anti-ZIKV small molecules are shown to be only active in in vitro cell assays. But the good news is that 20 of these compounds were evaluated in vivo, and three reached phase I clinical trials [99,142]. The current stage of anti-ZIKV drug discovery demands novel approaches to identify new inhibitors which may provide completely new class of antiviral drugs including Zika drugs. The approaches should include knowledge-based drug repurposing [143], RNA interference [144], long noncoding RNAs [145], interfering peptides [146,147], compounds targeting viral RNA [148,149,150], and computer-assisted molecular design including pharmacophore modeling [93]. Repurposing approach of old drugs allows to focus on certain types of candidates, such as kinase inhibitors and antimalarial drugs. Literature searching should include hidden or overlooked candidates. For example, the EGR1 target derived cell penetrating peptide was initially identified as an HIV inhibitor [151] and had been overlooked. However, later on this peptide was found to exhibit anti-ZIKV activity in Vero cells at EC50 of 0.1–0.4 μM (unpublished data). Natural products derived from plants have been successfully used to treat a variety of vector borne diseases, such as malaria. Natural products fundamentally differ from synthetic compounds in both chemical scaffolds and mechanisms of 370 action, and have great potential for the development of antiviral agents [152,153]. Oxidative stress has also been reported to play an important role in the pathogenesis of both RNA and DNA viruses [154]. Compounds known to exert oxidative stress may also be tried against ZIKV.

Pursuit of repurposing strategy on known drugs against ZIKV revealed that a few antimalarial drugs do possess anti-ZIKV activity in vitro [155,156,157,158] and in vivo [159,160]. With a similar objective, we recently reported [41] an in silico modeling study by developing an “interaction pharmacophore model” from stereoelectronic properties of 12 known antimalarial aminoquinoline derivatives using quantum chemical (QC) calculations. By utilizing the “interaction pharmacophore model” for similarity analysis on aminoquinoline derivatives found in the published literature [85], we identified three antimalarial aminoquinolines, Quinacrine (QC), Mefloquine (MQ), and GSK369796 active against ZIKV in in vitro assay [41]. Activities against ZIKV of the three aminoquinolines were found to be: EC50 values: 2.27 ± 0.14 mM (for QC); 3.95 ± 0.21 mM (for MQ); and 2.57 ± 0.09 mM (for GSK369796). A large extended negative potential region with a large hole over the N atom containing the aromatic ring in the compounds appear to be responsible for potent anti-ZIKV activity of the compounds. Thus, a strong H-bond acceptor site, a moderately strong H-bond donor site and a large distribution of hydrophobic area in the aminoquinolines may be used as pharmacophore template for virtual screening of other databases to identify new potential anti-ZIKV antimalarial compounds. However, both in silico and experimental studies would be required on antimalarial compounds for actual consideration of “repurposing” of old antimalarial drugs as potential anti-ZIKV drugs.

Apart from the above targets, other novel host-based targets, such as host glycosidase and novel iminosugar targets were also explored. For example, the two targets UV-4 and UV-5 have shown to reduce filovirus infections in vitro and in vivo, including DENV infection [160]. Therefore, these novel host-based targets have the potential for developing broad-spectrum antiviral drugs including anti-ZIKV drugs. In addition, bioinformatic approach generated from targets like siRNA [144] and interacting peptides [147] could be useful for ZIKV drug discovery.

In silico structural-bioinformatics-based methodologies and frameworks have also been developed for the design of ZIKV antibodies. One of these methodologies deals with RosettaAntibodyDesign (RAbD) samplings of diverse sequence, structure, and binding space of antibodies to antigens in customizable procedures for the design of broad range antibodies with different applications [161]. This method approaches from antibody sequences and structures by capturing structures from a diverse set of the canonical clusters and complementarity known as CDRs. Accuracy determinations includes sampling of the features using Monte Carlo design procedures [162].

Additional in silico approaches include de novo design of humanized monoclonal antibody regions by targeting a specific antigen epitope. Since monoclonal antibodies as therapeutic agents are increasingly becoming important for the treatment of cancers, infectious diseases, and autoimmune disorders, the approach may also be worthwhile for developing anti-ZIKV agents. A promising work to this end was reported recently by developing a technique known as optimal method for antibody variable region engineering with the OptMAVEn-2.0 software [163].

Most of the anti-ZIKV compounds identified so far have shown only low to moderate in vitro efficacy and rarely in vivo efficacy with clinical data. Therefore, search and testing for new compounds should continue till a suitable less toxic and effective anti-ZIKV drug is found. Although no anti-ZIKV drug has yet been discovered by pharmacophore modeling, many studies have indicated that this approach should provide a robust cornerstone to the medicinal chemists for synthesis of multifunctional inhibitors that will eliminate cross-resistance and toxicity as well as improve patient adherence [105].

Since screening for compounds by pharmacophore is essentially a knowledge-based approach, it implicitly provides certain information about the nature of the receptor-binding site or the nature of the ligand expected to bind at the active site. Therefore, it can serve as a complimentary tool to high-throughput screening (HTS) for rapid and effective experimental assay from a large pool of compounds in a short time. This approach thus cannot only provide useful insights about active site binding interactions but also can facilitate interdisciplinary discussions with medicinal chemists, synthetic chemists, toxicologists, and pharmacologists for a successful anti-ZIKV drug discovery program [164].

## 5. Discussion

*“Those alone are wise who act after investigation.”*

*Charaka, Sutrasthana 10:5*

*“We haven’t got the money, so we’ve got to think.”*

*Ernest Rutherford*

This review article on the Zika virus covers the following stages of modern computer-assisted approaches in the characterization of Zika virus, risk assessment, and the prevention as well as mitigation of the infectious epidemic/pandemic:

(a) *External characterization*: modelling of disease spread mechanisms, vectors, and reservoirs of ZIKV (and emerging pathogens in general);
(b) *Internal characterization:* bioinformatics-based sequence comparison methods to compare the genetic material of emerging strains/pathogens with the existing ones;
(c) *Vaccine design:* computer-aided vaccine design methods that are applied on important sequences detected in the previous step to produce potential vaccine candidates for quick synthesis/production/testing;
(d) *Drug design:* computer-assisted methods to propose and validate novel therapeutic molecules that may be precursors of new anti-Zika drugs or repurposing of already approved drugs.

These four stages provide a holistic and proactive step-by-step approach toward combating ZIKV and potential future pathogens using computational methods.

### 5.1. Quantitative Characterization of ZIKV Infection

In the realm of mathematical epidemiology discussed in Section 4.1 above, Daniel Bernoulli [165] pioneered the application of mathematical tools in epidemiology in 1766. This was further enriched by the extensive research of Robert May, among others, in the 20th century [166,167]. To this day, the analysis of field data using variants of the SIR differential equations (Equations 1a-1c) given in Section 5.1 gives us the ability to predict the trajectory of an infection process in a compact manner [167]. In recent years, several Zika-specific extensions of the SIR model have been explored for the prediction of ZIKV infection [30,31,32,33,34,35,36].

Besides SIR-type models, public health management in the case of emergence of epidemics/pandemics can benefit from the application of discrete mathematics models of the isolated sequences and associated computational tools. Because viruses are very complex (e.g., ZIKV has around 11,000 bases in its RNA), the sequences can be converted to real numbers or vectors using methods of graph theory, information theory etc. [9,17,75,76,84,168,169,170,171]. Such quantitative descriptors, either alone or a collection of them, can be used for viral sequence comparison. These methods are known as “alignment-free” techniques, as opposed to the protocols like BLAST [69,172,173], which are based on alignment of the bases of the sequences to be analyzed.

### 5.2. Sequence Comparison Methods

Section 4.3 above discusses (Appendix A; Table 4 and Table 5) the results on applications of alignment-free methods to the Zika virus based on the structure (sequence)-property similarity principle [79] using standard bioinformatics tools like PCA and SOM. In the PCA analysis (Appendix A), the first six principal components (PCs) explain more than 99% of the variance in the data. In one of our earlier studies with 3692 chemicals and 90 calculated molecular descriptors we found that first ten PCs explained more than 92% of the variance in the data [174]. This discriminatory power of the orthogonal variables or PCs derived from the calculated ZIKV RNA sequence descriptors is evident from the PC1 versus PC2 plot in Figure 4 which can clearly separate the African ZIKV sequences from those from Asia and South America. With the refinement of descriptor space by the addition of other indices, such analyses will be improved and will be able to place a novel or newly emerging viral sequence close to a similar set of objects and help us to make a preliminary assessment of their potential in causing an epidemic/pandemic. The clustering of the sequences using the SOM approach (Figure 5) is even more interesting. Here ZIKV sequences from various countries and regions are clearly separated from one another. It seems that the descriptors, which count the neighbors of individual bases, sufficiently describe the DNA/RNA sequences and are suitable for chemometrical analysis.

Similar results can be seen in Table 4 in the case of alignment-free descriptors like *gR* in a basic 2D graphical representation; a sequence alignment model also shows that the three sets of data are clearly separated (Figure 2), although not many sequences can be aligned at a time because of computational issues. However, what these results imply is that the space of numerical descriptors fairly well reflects the changes in base distributions in the viral sequences and can be adapted for monitoring sequence shifts and drifts, some of which may herald increased virulence and pathogenicity.

### 5.3. Computer-Assisted Vaccine Design

Development of vaccines against ZIKV is being attempted. But as of now, no vaccine is available for use in Zika Virus infected patients. Traditional vaccine development methods with its long history of successes is still a time-consuming and costly affair, poorly suited to the current circumstances where viral epidemics are more frequent and affects large swathes of the human population within a short time. Peptide vaccines developed ab initio against newly emerging viruses and epidemics, as mentioned in Section 4.2, hold great promise in the fight to control and stem the spread of the disease. These are comparatively easy to design and manufacture, store and transport, that traditional vaccines like live attenuated or inactivated vaccines come up short on. While peptide vaccines have been in use in veterinary diseases for some time, and there have been many trials as recorded in ClinicalTrials.gov website of the NIH, no license for human applications has been attained by any potential peptide vaccine to date. Some of the hurdles to be overcome include the need for adjuvants and carrier proteins [63], the careful regulation of stimulus for T-cytotoxic cells [64], guard against loss of native configuration, among others. On the other hand, the possibility of making small changes in the peptide composition to retain its effectiveness while at the same time conforming to the immune profile of the local populations promise more effective vaccination against emerging viral epidemics and pandemics. While quantitative and computational initiatives has been made to assist in this agenda, and designs for a Zika virus vaccine have already been advanced using these procedures [62], a more robust approach and applications of AI can be expected to boost precision vaccine design and further benefit future efforts in this sphere.

### 5.4. Anti-Zika Drug Discovery

Since a 3D structure of ZIKV protein is now known, discovery for potential anti ZIKV drugs can be accomplished through a combination of structure-based design, ligand docking, and pharmacophore modeling from literature known active compounds and virtual screening of compound databases. However it is important to note that even though the 3D structure of ZIKV proteins are known, specific features, such as polarization factors, effects of hydrogen bonding arising from specific patterns of solvation in water in the common ATP-binding pockets and dynamic target-inhibitor interactions may not be detectible by x-ray crystallography. Thus to account for such interactions, quantum chemical methods along with molecular dynamics should be better for the identification of potent anti-ZIKV ligands prior to experimental validations. In addition, alternative targets of established 3D protein structures implicated for ZIKV proteins should also be explored for ZIKV drugs. Although pharmacophore modeling has demonstrated many successes in the rational approach for discovery of potential therapeutics solely from structure-activity relationships of known bioactive compounds, without testing for efficacies of identified compounds no compound should be promoted. First in vitro testing followed by in vivo if found promising is absolutely necessary. In summary, it may be commented that for accomplishing a successful ZIKV drug discovery program, the research team should have good medicinal and synthetic chemistry support along with support from molecular biology and modeling groups for a rapid lead optimization.

Both peptide vaccine design and anti-ZIKV drug discovery involve high throughput in-silico, medium throughput in vitro, and low throughput in vivo methodologies. The availability of high-speed computers and useful software tools allow us to carry out numerous in silico experiments which act as a “decision support system” to the more expensive and time-consuming subsequent steps of in vitro and in vivo methods. This collaborative approach between traditional laboratory experiments and machine learning based prioritization of chemicals has the potential to judiciously use the limited resources available to the vaccine/drug designer. To this end, an interdisciplinary approach that leverages expertise of experts from different domain like epidemiology, machine learning, pharmacology etc., is needed to generate useful insights.

As observed by Virchow [27] and borne out by accumulated public health data over the ages, pathogens causing epidemics and pandemics are emerging and reemerging at random. Recently, specifically on 17th July 2019, the WHO declared the Ebolavirus disease (EVD, or Ebola) outbreak in the Democratic Republic of the Congo (DRC) a Public Health Emergency of International Concern (PHEIC) [175]. We believe that the four-pronged approach developed by us [28] and discussed in more details in this article may provide a starting point to develop “generic approaches” for the management of emerging global pathogens in an effective manner.

## Figures and Tables

**Table 1:** Structure of a typical flavivirus genome including UTRs. Typical sequence length data taken from ZIKV isolate ZIKV/*H. sapiens*/Brazil/PE243/2015 (GenBank Locus ID KX197192), complete genome. E: envelope; NS: non-structural; nt: nucleotide; prM/M: pre-membrane/membrane; UTR: untranslated region.

Gene	Sequence Span, nt	Sequence Length	Protein/Biological Function
5′-UTR	1–107	107	Encodes regions essential for genome cyclization/replication.
Capsid	108–473	366	Virion structure.
prM/M	474–977	504	prM forms heterodimers with E to form immature virion. prM then cleaved and mature virions formed with M.
E	978–2489	1512	Viral entry into host cell.
NS1	2490–3545	1056	Viral replication, immune evasion, genome synthesis.
NS2A	3546–4223	678	Transmembrane protein, part of replication complex; assembly/secretion of virus particles.
NS2B	4224–4613	390	Cofactor for proteinase domain of NS3; proteolytic processing.
NS3	4614–6464	1851	Protease/helicase.
NS4A	6465–6914	381	Viral RNA replication and amplification.
2K	6846–6914	69	Peptide generated by cleavage at the N terminus of the NS4B signal sequence.
NS4B	6915–7667	753	Facilitates viral replication complexes; counteracts innate immune responses.
NS5	7668–10376	2709	Methyltransferase; RNA-dependent RNA polymerase.
3′-UTR	10380–10807	427	Facilitates viral replication and translation.

**Table 2:** The pre-epidemics period of the Zika virus after its detection in 1947. *A. africanus*: *Aedes africanus*; ZIKV: Zika virus.

Year	Country	Remarks
1947	Uganda	First isolation and identification of ZIKV. Found in rhesus monkey, R766, caged in Zika forest.
1948	Uganda	Detected in *A. africanus* mosquitoes. Found in Zika forest.
1951	Nigeria	First instance of ZIKV antibodies in human blood, found in children.
1952	Uganda, Tanganyika	First human cases of ZIKV infection detected.
	India	ZIKV antibodies found in human blood.
1953	Malaya, North Borneo, Philippines	ZIKV antibodies found in residents.
	Nigeria	ZIKV infection detected in three persons.
1954	Egypt, Vietnam	ZIKV antibodies found in few residents.
1955	Nigeria	ZIKV antibodies found in human blood.
1957	Mozambique	ZIKV antibodies found in human blood.
1958	Uganda	Two strains of ZIKV found in *A. aegypti* mosquitoes in Zika forest.
1960	Angola	ZIKV antibodies found in indigenous residents.
1961–1962	Central African Republic	ZIKV antibodies found in human blood.
1961–1964	Ethiopia	ZIKV antibodies found in human blood.
1962	Senegal	ZIKV antibodies found in human blood.
1963–1964	Central African Republic, Burkina-Faso	ZIKV antibodies found in human blood.
1963–1965	Ivory Coast	ZIKV antibodies found in human blood.
1964	Uganda	First confirmation that ZIKV causes human disease. Clinical features reported.
1964–1965	Guinea-Bissau	ZIKV antibodies found in human blood.
1964–1966	Togo, Cameroon	ZIKV antibodies found in human blood.
1965	Niger	ZIKV antibodies found in human blood.
1965–1967	Nigeria	ZIKV antibodies found in human blood.
1967	Benin, Gabon, Liberia	ZIKV antibodies found in human blood.
1966–1967	Uganda, Kenya, Somalia, Morocco	ZIKV antibodies found in human blood.
1967–1969	Uganda	ZIKV antibodies found in human blood.
1968	Kenya	ZIKV antibodies found in human blood.
1969–1972	Nigeria	ZIKV antibodies found in human blood.
1969	Malaysia	ZIKV found in *A. aegypti* mosquitoes.
1969–1983	Indonesia, Malaysia, Pakistan	ZIKV found in mosquitoes. Sporadic human infections.
1970	Nigeria	ZIKV antibodies found in human blood.
1971–1972	Angola	ZIKV antibodies found in human blood.
1972,1975, 1988,1990	Senegal	ZIKV antibodies found in human blood.
1979	Central African Republic	ZIKV antibodies found in human blood.
1980	Nigeria	ZIKV antibodies found in human blood.
1984	Uganda	ZIKV antibodies found in human blood.
1996–1997	Malaysia	ZIKV antibodies found in human blood.
1999	Ivory Coast	ZIKV antibodies found in human blood.

**Table 3:** A brief summary of ZIKV epidemics, 2007 to present. Data summarized from [18,19].

Year	Country	Remarks
2007	Yap Island, Micronesia	First outbreak reported in humans.
2008	Senegal	First reported case of traveler infected in Senegal returning to home country and passing infection through sexual contact.
2010	Cameroon	ZIKV antibodies found in human blood.
2010–2015	Cambodia, Indonesia, Malaysia, Philippines, Thailand, Maldives	Mosquito transmission of ZIKV in these countries to travelers who then carried the infection to their home countries.
2011–2014	French Polynesia	Second reported outbreak of ZIKV infections. Connection with microcephaly and neurological disorders established later.
2013–2014	Chile, Cook Islands, New Caledonia	ZIKV outbreak.
2013	Tahiti	ZIKV isolated from patient’s semen showing sexual transmissibility.
2014	Zambia	ZIKV antibodies found in human blood.
2015 April/May	Brazil, Bahia state	National Reference Laboratory, Brazil confirmed, by PCR, ZIKV infections, for the first time in the Americas.
2015 July	Brazil	Zika cases confirmed by laboratory tests in 12 states. Neurological disorders associated with prior viral infections detected primarily in the Bahia region.
2015 October	South America	Colombia, Republic of Cabo Verde report confirmed cases of ZIKV infections. Brazil reported unusual increase in the number of cases of neonatal microcephaly.
2015 November	Central and South America	Brazil reported 141 suspected cases of microcephaly and declared a national public health emergency. Brazil reported detection of ZIKV in amniotic fluid of fetuses with confirmed microcephaly. Suriname, Panama, El Salvador, Guatemala, and Paraguay confirmed cases of ZIKV infection. The Pan American Health Organization and WHO issued an epidemiological alert.
2015 November	Mexico	Three cases of ZIKV infection confirmed by PCR.
2015 November	French Polynesia	Retrospective analysis reveals unusually large number of central nervous system malformations in fetuses and infants in 2014–2015.
2015 December	Central and South America	Honduras, French Guiana, and Martinique reported confirmed cases of ZIKV infections.
2015 December	Puerto Rico	First confirmed case of Zika infection reported.
2016	Maldives	Finnish national working in Maldivestested positive for Zika after return to Finland.
2016 January	Americas	Guyana reported the first PCR-confirmed case of locally acquired Zika infection. Ecuador, Bolivia, Barbados, Haiti, Dominican Republic, Nicaragua, Curacao and Jamaica reported the first confirmed cases of Zika infections. First case of Zika in St. Martin reported.
2016 January	US Virgin Islands	First confirmed case of Zika in St. Croix reported.
2016 February	Americas	First confirmed case of ZIKV infection in Chile reported. First case of sexually transmitted Zika infection in Texas, USA reported.
2016	Various countries	Angola, Antigua, British Virgin Islands, Trinidad and Tobago, Guadulope, Fiji, Marshall Islands, Papua New Guinea, and other countries report first cases of ZIKV infections.
2016	Singapore	ZIKV infection reported.
2016/2017	India	Three cases of ZIKV infection reported in Ahmedabad.
2017	Singapore	Several cases of locally transmitted ZIKV confirmed.

**Table 4:** *gR* values of all sequences of the Zika virus genome with 10,272 identified bases and selected hosts each, continent wise.

Location	No of Seqs	Average *gR*	Std Dev	Change	Hosts
Africa	7	100.80	0.58	-	*Aedes africanus, A. taylori*
Asia	106	89.08	3.95	−11.63%	*Homo sapiens*
South America	103	85.92	3.31	−4.55%	*Homo sapiens*

**Table 5:** The objects (virus samples) with Mahalanobis distance (*d*) larger than 180 (Average + 2*Standard deviation).

Continent	*d*	Locus ID	Year	Country	Host
Africa	83.1238	KF268949	1980	Central African Republic	*Aedes opok*
	226.8671	KF383115	1968	Central African Republic	*Aedes africanus*
	187.0571	KF383116	1968	Senegal	*Aedes luteocephalus*
	204.2905	KF383118	2001	Senegal	*Aedes dalzieli*
Asia	219.0294	KY241697	2016	Singapore	*Homo sapiens*
	258.6828	KY241700	2016	Singapore	*Homo sapiens*
	224.1343	KY241704	2016	Singapore	*Homo sapiens*
	286.1132	KY241766	2016	Singapore	*Homo sapiens*
	261.6707	MK238035	2018	India	*Homo sapiens*
	261.6707	MK238038	2018	India	*Homo sapiens*
South America	220.3336	KY559005	2018	Brazil	*Homo sapiens*
	261.9161	KY559027	2018	Brazil	*Homo sapiens*
	242.1343	KY785427	2018	Brazil	*Homo sapiens*
	264.0801	KY785429	2018	Brazil	*Homo sapiens*
	185.0375	KY785433	2018	Brazil	*Homo sapiens*
	283.8677	KY785456	2018	Brazil	*Homo sapiens*
	294.1674	MH882537	2018	Brazil	*Homo sapiens*

**Table 6:** Anti-ZIKV compounds having the potential to be drugs.

Compounds	Derivatives	Reference
Chloroquine	Derivatives particularly at the C-4 position of *N*-(2-arylmethylimino)ethyl-7-chloroquinolin-4-amine derivatives	[98]
Quinacrine (QC), Mefloquine (MQ), and GSK369796	Antimalarial aminoquinoline derivatives	[41,87]
PHA-690509	Cyclin dependent kinase (CDK) inhibitor	[99]
Lapachol, HMC-HO1α and Ivermectin	Hybrid drugs against co-infections of ZIKV, dengue and chikungunya	[100]
20-Cmethylated nucleosides	Inhibitors of RNA-dependent RNA polymerase (RdRp)	[101]
NS3 inhibitors	Covalent inhibitors of a viral protein and anti-Toll-like receptor molecules	[102]
FDA-approved drugs	In vitro screening of 774 compounds led to twenty compounds that were found to reduce ZIKV infection	[93]
NIH clinical library of compounds	By screening 725 chemically diverse compounds from the library, 22 compounds were reported to have potent anti-ZIKV activity of which five were found promising. These are Lovastatin (Pubchem CID: 53232), 5-Fluorouracil (Pubchem CID: 3385); 6-Azauridine (Pubchem CID: 5901); Palonosetron (Pubchem CID: 6337614) and Kitasamycin (Pubchem CID: 44634697).	[103]
A limited proprietary library of small organic compounds	Anti-ZIKV activity through screening and confirming potent anti-ZIKV activity in in vitro plaque assay	[104]
NITD008	A type of nucleoside adenosine analog	[105]
Warfarin and a few similar structural analogues	Inhibitors of dimerization of Axl receptor (a tyrosine kinase)	[106]
Nonsteroidal anti-inflammatory drugs (NSAIDs), including aspirin, ibuprofen, naproxen, acetaminophen, and lornoxicam, potently inhibited the entry of Zika virus Env/HIV-1-pseudotyped viruses	Inhibited replication of wild-type ZIKV both in cell lines and in primary human fetal endothelial cells. Interestingly, the NSAIDs exerted this inhibitory effect by potently reducing the expression of AXL, the entry cofactor of ZIKV. Further studies showed that the NSAIDs downregulated the prostaglandin E_2_/prostaglandin E receptor 2 (EP2)/cAMP/protein kinase A (PKA) signaling pathway and reduced PKA-dependent CDC37 phosphorylation and the interaction between CDC37 and HSP90, which subsequently facilitated CHIP/ubiquitination/proteasome-mediated AXL degradation.	[107]
Nanchangmycin	Envelope glycoprotein inhibitor	[90]
Temoporfin, NSC157058	NS2B-NS3protease inhibitors	[108]
Suramin	NS3 polymerase inhibitors	[109]
Sofosbuvir, 2′-C-ethynyl-UTP and DMB213	NS5 polymerase inhibitors	[110]
Sinefungin	NS5 methyltransferase inhibitor	[111]
6-azauridine and 5-fluorouracil	Pyrimidine biosynthesis inhibitors	[93,95]
Lovastatin and Mevastatin	HMG-CoA reductase inhibitor	[112]
BCX4430	An adenosine nucleoside analog, functions as a selective inhibitor of viral RNA-dependent RNA polymerase (RdRp). It was found that BCX4430 had EC_50_ values in the range 3.8–18.2 μg/mL in vitro, with favorable selective index (SI) values. In a mouse model of ZIKV infection (300 mg/kg/d), treatment with BCX4430 showed promising results. The protective effect of BCX4430 was observed to continue for 24 h even after virus challenge.	[113]

**Figure A1:**
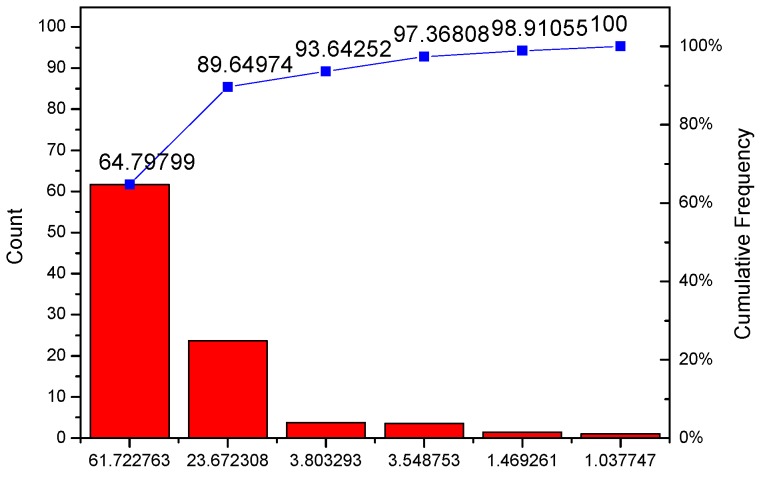
Scree plot of first six PCs, carrying over 99.9% of variance.

## Appendix A. Technical Details of PCA Analysis

The data for this investigation can be viewed as *n* = 310 vectors (sequences) in *p* = 53 dimensions (computed sequence descriptors). Over 93% of sequences have 10272 bases. Details on the source of data as well as pre-processing steps are available in [82]. These data can be represented by a matrix X which has 310 rows and 53 columns. Thus, each sequence is represented by a point in ${\mathbb{R}}^{53}$. If each sequence could be represented in ${\mathbb{R}}^{2}$, one could plot and investigate the extent to which similar sequences are situated near each other according to the two descriptors. In ${\mathbb{R}}^{53}$ such simple analysis is not possible. However, since many computed descriptors are strongly correlated with one another, 310 points in ${\mathbb{R}}^{53}$ will lie nearly on a subspace of dimension lower than 53. The method of principal component analysis (PCA) or the Karhunen–Loeve transformation is a standard linear method for reduction of dimensionality in such cases.

**Table A1:** The PCs with explained variance > 1%.

C	% Variance Explained
PC1	61.72276
PC2	23.67231
PC3	3.80329
PC4	3.54875
PC5	1.46926
PC6	1.03775

**Table A2:** Descriptor-wise loadings of the first six PCs. Bold numbers indicate significant loadings.

Descriptor	PC1	PC2	PC3	PC4	PC5	PC6
A	**0.37525**	−0.19154	−**0.30383**	−0.14333	−0.03459	−0.0214
C	**0.30264**	**0.30112**	0.02034	−**0.24198**	0.05169	0.08496
G	**0.45499**	0.08624	**0.27048**	0.08604	0.10882	−0.04221
T	**0.30835**	−0.05888	−**0.20629**	**0.32184**	-0.15941	0.00631
1. neigh.AA	0.08084	0.14915	−**0.32239**	0.06124	−0.0785	0.00945
AC	0.06785	0.01709	−0.01214	−0.18303	0.0477	−0.05584
AG	0.14214	−**0.20504**	0.05524	−0.05171	−0.00698	−0.07525
AT	0.09397	−0.15383	−0.02502	0.02867	0.00811	0.10061
CA	0.11279	−0.15156	0.12023	−0.04033	−0.08225	0.00573
CC	0.08502	0.05509	0.11682	−0.11607	−0.1074	−0.18759
CG	0.03336	**0.23813**	−0.04533	0.06401	**0.31908**	−0.02018
CT	0.07977	0.16021	−0.16937	−0.14672	−0.06374	**0.28541**
GA	0.15174	−**0.22587**	0.0435	−0.17946	0.07887	−0.05781
GC	0.0845	0.14153	0.01696	0.10111	0.03199	0.03393
GG	0.15024	0.18539	0.15847	0.11032	0.1174	0.05727
GT	0.08098	−0.01346	0.04668	0.05517	−0.11229	−0.07188
TA	0.04247	0.03678	−0.14869	0.01361	0.05463	0.02565
TC	0.07274	0.0886	−0.09937	−0.04316	0.08842	0.29342
TG	0.13751	−0.13156	0.10156	−0.03741	−**0.31189**	−0.00237
TT	0.06248	−0.05234	−0.06042	**0.38732**	0.01392	−0.30868
2. neigh.AA	0.09635	−0.09113	−0.18414	−0.01641	−0.01696	0.08857
AC	0.08684	−0.01636	−0.01001	−0.02046	**0.24963**	0.0758
AG	0.12814	−0.07697	0.12034	−0.0776	−0.09957	−0.17652
AT	0.07514	−0.00809	−**0.22651**	−0.02836	−0.15486	−0.00966
CA	0.06435	0.17475	−0.05956	0.06259	−0.12713	**0.20447**
CC	0.06424	0.17368	−0.03028	−0.03357	−0.01968	−**0.21684**
CG	0.08972	0.11317	0.0235	−**0.31158**	−0.06387	−0.04091
CT	0.09397	−0.15892	0.09194	0.04293	**0.27383**	0.13541
GA	0.14944	−**0.19937**	0.00492	−**0.24278**	0.00391	−0.15607
GC	0.0848	0.12145	0.00406	−0.07691	0.15546	−0.08149
GG	0.14281	0.1253	0.10387	**0.22558**	0.21362	0.09455
GT	0.09975	0.04099	0.15838	0.18232	−0.24926	0.10006
TA	0.07649	−0.07442	−0.06657	0.05378	0.11689	−0.15562
TC	0.08115	0.02246	0.06024	−0.10957	−0.32184	**0.30228**
TG	0.11086	−0.07303	0.02508	**0.25033**	0.07385	0.08249
TT	0.05183	0.0677	−**0.23082**	0.12703	−0.02029	−0.22091
3. neig.AA	0.11116	−0.08791	−**0.23494**	0.00908	0.02267	−0.02569
AC	0.08419	−0.12537	−0.00149	−0.14264	**0.23392**	0.04934
AG	0.12295	−0.03915	0.09917	−0.05518	−0.10449	0.08412
AT	0.07376	0.06077	−0.16567	0.04808	−0.16838	−0.12946
CA	0.07496	0.11183	0.11847	-0.0885	−0.16906	−0.14642
CC	0.08748	0.05298	0.09463	1.18E-4	−0.06922	0.22857
CG	0.09406	−0.01214	−0.03707	−0.06271	0.16529	0.07823
CT	0.05919	0.14918	−0.15259	−0.08828	0.13657	−0.08248
GA	0.12164	−0.16697	−0.05858	−0.08846	0.15677	0.08178
GC	0.08017	**0.40191**	0.01602	−0.02209	−0.10253	−0.04902
GG	0.17269	0.09535	0.17819	0.01763	0.05321	−0.28992
GT	0.10798	−**0.24128**	0.14189	0.18252	0.01912	**0.20649**
TA	0.07981	−0.04709	−0.12778	0.02289	−0.03166	0.06871
TC	0.06893	−0.02786	−0.0826	−0.07336	0.00371	−0.14839
TG	0.09038	0.04417	0.03068	0.19045	0.0128	0.08684
TT	0.08232	−0.02577	−0.02793	0.18266	−0.13476	0.00604
rG	0.05465	0.02002	**0.34324**	−0.0189	−0.07488	−0.03129
